# Cell death pathways and viruses: Role of microRNAs

**DOI:** 10.1016/j.omtn.2021.03.011

**Published:** 2021-03-19

**Authors:** Javid Sadri Nahand, Layla Shojaie, Seyed Amirreza Akhlagh, Mohammad Saeid Ebrahimi, Hamid Reza Mirzaei, Hossein Bannazadeh Baghi, Maryam Mahjoubin-Tehran, Nima Rezaei, Michael R. Hamblin, Vida Tajiknia, Neda Rahimian, Hamed Mirzaei

**Affiliations:** 1 Department of Virology, Faculty of Medicine, Iran University of Medical Sciences, Tehran, Iran; 2 Research Center for Liver Diseases, Keck School of Medicine, Department of Medicine, University of Southern California, Los Angeles, CA, USA; 3 School of Medicine, Shiraz University of Medical Sciences, Shiraz, Iran; 4 School of Medicine, Kashan University of Medical Sciences, Kashan, Iran; 5 Department of Medical Immunology, School of Medicine, Tehran University of Medical Sciences, Tehran, Iran; 6 Infectious and Tropical Diseases Research Center, Tabriz University of Medical Sciences, Tabriz, Iran; 7 Department of Medical Biotechnology, Faculty of Medicine, Mashhad University of Medical Sciences, Mashhad, Iran; 8 Research Center for Immunodeficiencies, Children’s Medical Center, Tehran University of Medical Sciences, Tehran, Iran; 9 Network of Immunity in Infection, Malignancy and Autoimmunity (NIIMA), Universal Scientific Education and Research Network (USERN), Tehran, Iran; 10 Laser Research Centre, Faculty of Health Science, University of Johannesburg, 2028 Doornfontein, South Africa; 11 Radiation Biology Research Center, Iran University of Medical Sciences, Tehran, Iran; 12 Department of Surgery, School of Medicine, Iran University of Medical Sciences, Tehran, Iran; 13 Endocrine Research Center, Institute of Endocrinology and Metabolism, Iran University of Medical Sciences (IUMS), Tehran, Iran; 14 Research Center for Biochemistry and Nutrition in Metabolic Diseases, Institute for Basic Sciences, Kashan University of Medical Sciences, Kashan, Iran

**Keywords:** cell death, apoptosis, autophagy, anoikis, viruses, microRNAs

## Abstract

Viral infections lead to the death of more than a million people each year around the world, both directly and indirectly. Viruses interfere with many cell functions, particularly critical pathways for cell death, by affecting various intracellular mediators. MicroRNAs (miRNAs) are a major example of these mediators because they are involved in many (if not most) cellular mechanisms. Virus-regulated miRNAs have been implicated in three cell death pathways, namely, apoptosis, autophagy, and anoikis. Several molecules (e.g., BECN1 and B cell lymphoma 2 [BCL2] family members) are involved in both apoptosis and autophagy, while activation of anoikis leads to cell death similar to apoptosis. These mechanistic similarities suggest that common regulators, including some miRNAs (e.g., miR-21 and miR-192), are involved in different cell death pathways. Because the balance between cell proliferation and cell death is pivotal to the homeostasis of the human body, miRNAs that regulate cell death pathways have drawn much attention from researchers. miR-21 is regulated by several viruses and can affect both apoptosis and anoikis via modulating various targets, such as PDCD4, PTEN, interleukin (IL)-12, Maspin, and Fas-L. miR-34 can be downregulated by viral infection and has different effects on apoptosis, depending on the type of virus and/or host cell. The present review summarizes the existing knowledge on virus-regulated miRNAs involved in the modulation of cell death pathways. Understanding the mechanisms for virus-mediated regulation of cell death pathways could provide valuable information to improve the diagnosis and treatment of many viral diseases.

## Graphical Abstract

**Figure:**
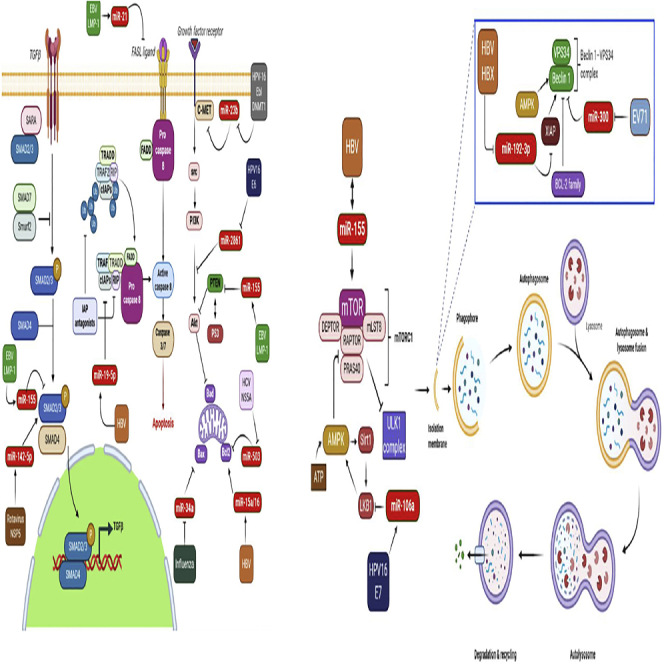

## Teaser

The miRNAs that are involved in the modulation of cell death pathways after virus infection show a degree of complexity and a variety of functions. Hence, these molecules can be used for further development of diagnostic and therapeutic techniques.

## Introduction

Virus infections have long posed a threat to human public health. Although virus-related deaths have sharply declined during the past century,^1^ the death toll is still relatively high. According to the World Health Organization (WHO), respiratory diseases linked to seasonal influenza lead to 650,000 deaths each year. Most recently, the ongoing pandemic of coronavirus disease 2019 (Covid-19) has officially been responsible for 1.6 million deaths so far (December 12, 2020). Additionally, viruses are indirectly involved in other lethal diseases; for instance, 15% of human cancer deaths are estimated to be associated with a viral infection.^2^

Over the whole range of viruses, from small RNA viruses (e.g., dengue virus [DENV]) to large DNA viruses (e.g., poxviruses), viruses uniquely manipulate the host cell machinery in order to facilitate the pathogenic process.^3^ Cell death pathways in particular have been shown to be strongly affected by viral infections. Because a marked imbalance between the rate of cell proliferation and cell death is associated with serious diseases such as cancer,^4^ dysregulation of cell death pathways, whether caused by a virus or other factors, is of great importance. Several factors associated with modulation of cell death pathways (e.g., autophagy-related genes^5^ and survivin^6^) have been shown to be dysregulated in virus-infected cells. One of the major molecular controllers of cell death is microRNAs (miRNAs), which can be affected by viral infections.

miRNAs are small non-coding RNAs that recognize and bind to the 3′ untranslated region (UTR) of messenger RNAs (mRNAs) that code for cellular proteins, in order to block the translation of the target protein or, to a lesser extent, degrade the mRNA.^7^, ^8^, ^9^, ^10^ miRNAs are key modulators of many fundamental cellular processes, including cellular metabolism, proliferation, and cell death in a variety of cell types, such as stem cells, immune cells, cancer cells, and especially virus-infected cells.^7^^,^^11^, ^12^, ^13^, ^14^ Hence, the virus-modulated expression of miRNAs that can result in the regulation of cell death pathways may explain the pathogenesis and lead to the possible treatment of viral diseases.

**Figure 1:**
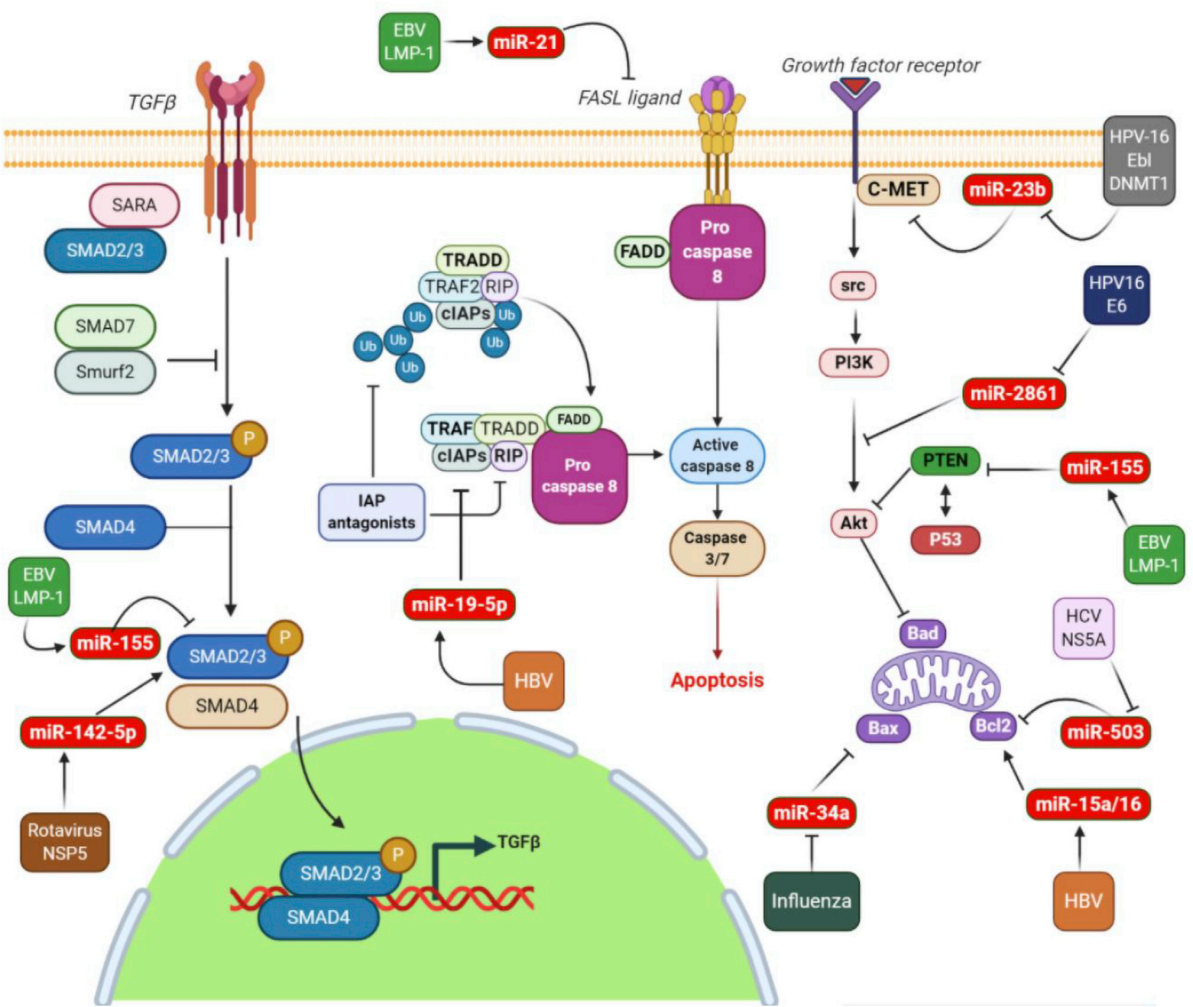
Indirect effects of viruses on the apoptotic pathway through deregulation of cellular miRNAs miR-2861 increased the apoptosis of cervical cancer cells through the PI3K/AKT pathway by targeting EGFR. HPV16 E6 is able to downregulate the miR-2861 expression level and can contribute to tumor development. Moreover, HPV-16 E6/DNMT1 can suppress miR-23b expression and contribute to apoptosis resistance through the blocking inhibitory effect of miR-23b on c-MET. EBV-LMP-1 protects B cell lymphoma from rituximab-induced apoptosis via miR-155-mediated Akt activation and upregulation of Mcl-1. LMP1 can increase miR-21 to promote the resistance of nasopharyngeal carcinoma cells to cisplatin-induced apoptosis by suppressing PDCD4 and Fas-L. HBV infection can also lead to the induction of apoptosis via the upregulation of miR-194-5p expression *in vitro*. miR-194-5p-mediated suppression of cFLIP expression strongly sensitizes HepG2 cells to undergo apoptosis in response to a physiological stimulus. The transforming growth factor β (TGF-β) signaling pathway, a major intercellular signaling pathway in mammalian cells, plays a key role in regulating many cellular processes, such as cell proliferation, differentiation, and apoptosis. TGF-β signaling is known to depend on the formation of Smad2/3-Smad4 transcription regulatory complexes. EBV LMP-2A inhibits the expression level of Smad2 through regulating miR-155-5p in gastric cancer cell lines and inhibits apoptosis. Rotavirus infection leads to up-regulation of TGF-β, which may lead to early apoptosis thus preventing virus progression. However, rotavirus counteracts this by upregulation of miR-142-5p. Rotavirus NSP5 upregulates miR-142-5p, which targets several components of the TGF-β signaling pathway. The mitochondrial pathway of apoptosis is dependent on the BCL-2 (B cell CLL/lymphoma 2) family of proteins for the efficient release of pro-apoptotic factors from the mitochondrial intermembrane space. The BCL-2 family is divided into three groups based on their primary function: (1) anti-apoptotic proteins (BCL-2, BCL-X_L_, BCL-W, MCL-1, BFL-1/A1); (2) pro-apoptotic pore formers (BAX, BAK, BOK); and (3) pro-apoptotic BH3-only proteins (e.g., BAD, BID, BIK, BIM). miR-503 and miR-15a/16 can target the 3′ UTR of Bcl-2 and inhibit hepatocellular carcinoma cell growth. However, HCV NS5A and HBV mRNA decrease miR-503 and miR-15a/16 expression, respectively, and increase Bcl-2 expression, which leads to a decrease in apoptosis.

**Figure 2:**
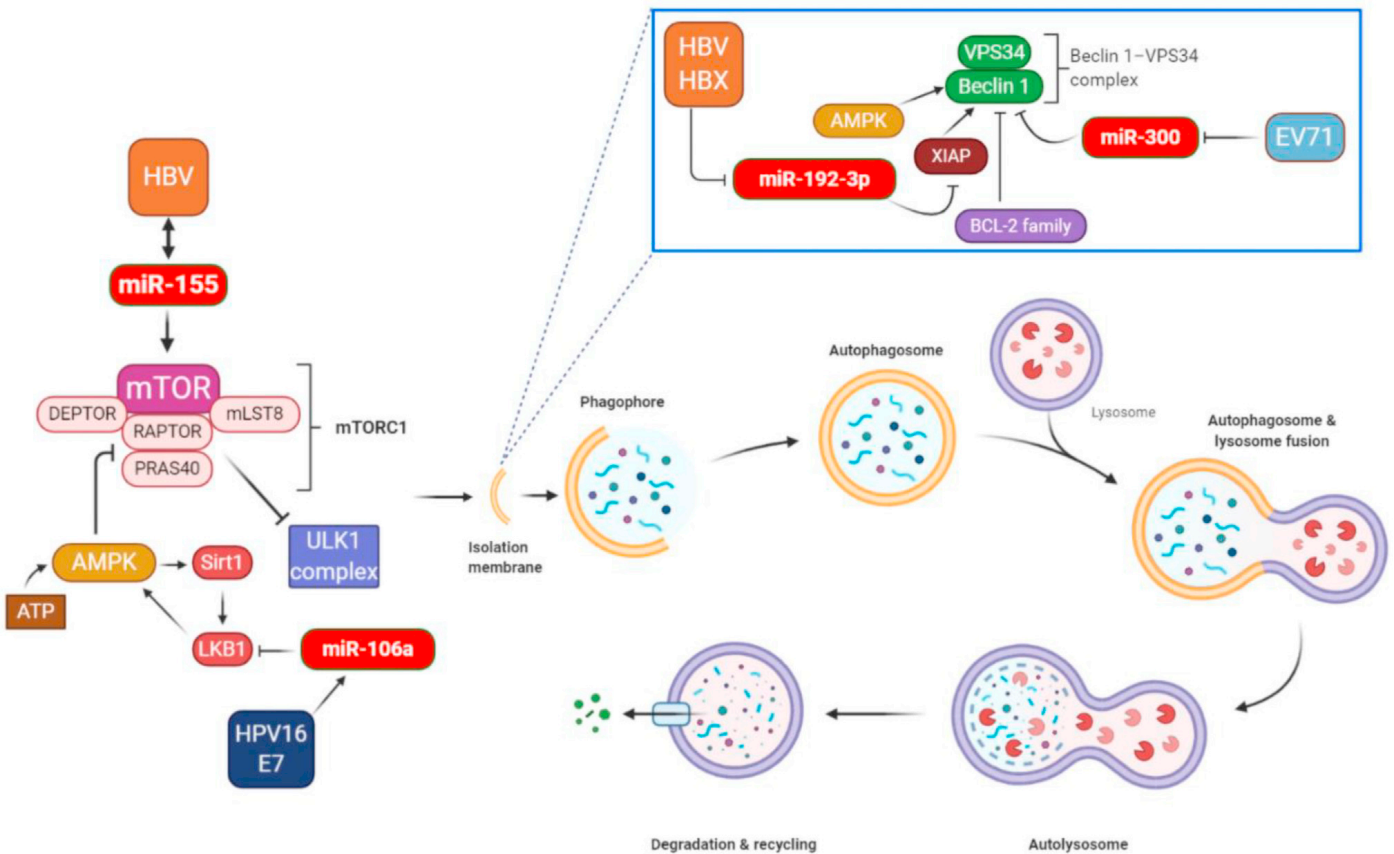
Indirect effects of viruses on the autophagy pathway through deregulation of cellular miRNAs Autophagy involves the spatially and temporarily coordinated activation of multiple molecular components, including the ULK1 (UNC-51-like kinase 1), FIP200 (FAK family kinase-interacting protein of 200 kDa), and ATG13–ATG101 complex, which is functionally coupled to the negative autophagy regulator, mTOR complex 1, and initiates autophagy. The lipid kinase vacuolar protein sorting 34 (VPS34)-Beclin-1 complex is usually inactivated by anti-apoptotic proteins from the BCL-2 family, but when it is activated it drives the nucleation of the isolation membrane.^15^ The AMP-activated protein kinase (AMPK)-mammalian target of rapamycin (mTOR) signaling pathway is well known to be associated with autophagy, and AMPK can promote the initiation of autophagy. Activation of AMPK can result in the inhibition of mTOR, which is commonly activated in malignant cells.^16^ miR-106a is upregulated by the HPV-16 E7 oncogene and stimulates cell proliferation and suppresses autophagy by targeting LKB1 via the AMPK-mTOR pathway in HPV-16-associated cervical cancer. Autophagy contributes to HBV replication, as confirmed by observations that autophagy inhibition strongly impaired replication of HBV in liver cells. The expression level of miR-155 was upregulated in HBV-infected cells, and miR-155 reinforced HBV replication by affecting the SOCS1/Akt/mTOR-autophagy axis. HBV also promoted autophagy through the miR-192-3p-XIAP axis, which is important for HBV replication *in vitro* and *in vivo*. Beclin-1 is a key autophagy-promoting gene in the early phase of autophagosome formation. It has been shown that EV71 infection induced autophagosome formation through the reduction of cellular miR-30a, which led to the inhibition of Beclin-1.

Viruses can affect cell death pathways by the deregulation of miRNAs, leading to interference with apoptotic and autophagic mechanisms, as shown in Figures 1 and 2. In the present review we summarize the effects of viruses on various miRNAs involved in the regulation of different cell death pathways, including apoptosis, autophagy, and anoikis.

### Apoptosis

The apoptosis pathways differ between normal cells and cancer cells and even between different cancer cell lines, making the design of therapeutic approaches based on apoptosis induction somewhat challenging.^17^^,^^18^ Therefore, these studies, which were fully focused on apoptosis, have been recently expanded to cover other cell death pathways, i.e., autophagy and anoikis.^19^ Exploring the cell death pathways and their mechanistic regulation may allow advances to be made in the diagnosis and treatment of several diseases.

Derived from the Greek root “apo” plus “ptosis,” meaning “falling off” (such as leaves falling off a tree),^20^ the term apoptosis refers to programmed cell death type I that has been long regarded as the main mechanism of controlled cell death.^21^ Apoptotic cell death is characterized by certain morphological features and some enzymatic processes occurring within the cell, which are designed to allow the removal of unneeded or defective cells without any significant risks for the surrounding tissues.^20^

**Figure 3:**
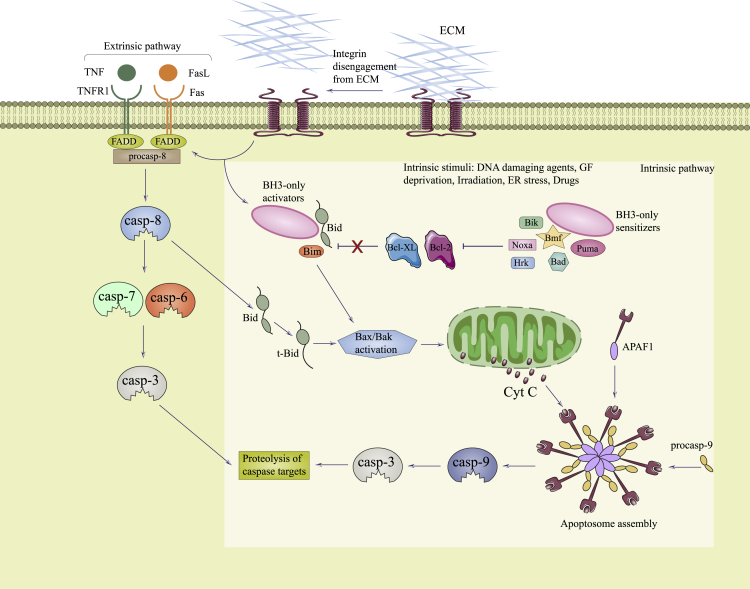
Comparison of apoptosis and anoikis The lack of ECM contact or the engagement with inappropriate ECM leads to the activation of anoikis either by death receptors (extrinsic pathway) or mitochondria (intrinsic pathway).

Researchers have divided apoptotic pathways into two broad classes, the intrinsic (mitochondrial) pathway and the extrinsic pathway (i.e., death receptor and the perforin/granzyme pathways) (Figure 3). Therefore, the extrinsic signaling pathway involves the tumor necrosis factor (TNF) receptor gene superfamily, and the intrinsic signaling pathway involves many components that can have a negative or positive effect on apoptosis. Apoptosis can be triggered by negative signals arising from the lack of certain growth factors, cytokines, and hormones. The mitochondrial membrane is influenced by such changes and thus the mitochondrial apoptosis pathway is activated. The control and regulation of mitochondrial apoptosis requires a balance between anti-apoptotic as well as pro-apoptotic members of the B cell lymphoma 2 (Bcl-2) protein family. Additionally, the inhibitor of apoptosis (IAP) family consisting of baculoviral IAP repeat-containing (BIRC) proteins has been found to be a major regulator of apoptosis, because these proteins affect both the intrinsic and extrinsic pathways.^22^

Both of these initial pathways (intrinsic and extrinsic) initiate the executioner (secondary) pathway of apoptosis through different, but related, routes. Both initial and executioner pathways of apoptosis are highly dependent on a family of cysteine-aspartic acid proteases called caspases. Caspases are divided into two groups, i.e., initiator or executioner, according to the pathway in which they participate.^23^^,^^24^ After detection of cell damage, procaspases are converted into the active initiator caspases (caspase-8 from the extrinsic pathway, and caspase-9 from the intrinsic pathway), which, in turn, activate the executioner caspases (caspase-3, -6, and -7). Once activated, the executioner caspases embark on a consecutive series of steps that culminate in DNA fragmentation. These steps include endonuclease activation, degradation of the nuclear proteins and cytoskeleton, protein cross-linking, the expression of ligands designed to attract phagocytes, and the formation of apoptotic bodies.^24^^,^^25^ The apoptotic bodies, which contain the potentially pathogenic remnants of cellular contents, will be further removed mainly by macrophages.^20^

Normal levels of apoptosis are required not only for cellular and organ homeostasis, but can also inhibit carcinogenesis.^26^^,^^27^ Apoptosis plays a major role in modulating multiple cellular processes such as the appropriate function and development of the immune system, hormone-regulated atrophy, embryonic development, cell recycling, and cell death in response to toxic stimuli.^24^ Activation of apoptosis irreversibly triggers efficient and rapid cell death through mitochondrial membrane permeabilization and caspase activation.^28^^,^^29^ The downregulation of apoptosis in cancer cells not only increases the proliferation rate, but it prolongs the survival time of the cancer cells through multiple pathways. Moreover, resistance of the malignant cells to apoptosis leads to several consequences, such as oncogene activation, metabolic stress, lack of blood supply, nutrient and growth factor depletion, tolerance to hypoxia, and failure of treatment interventions.^28^^,^^30^ In addition to cancer cells, virus-infected cells such as those infected with hepatitis B virus (HBV)^31^ or Epstein-Barr virus^32^ show resistance to apoptosis activation.

### Autophagy

**Figure 4:**
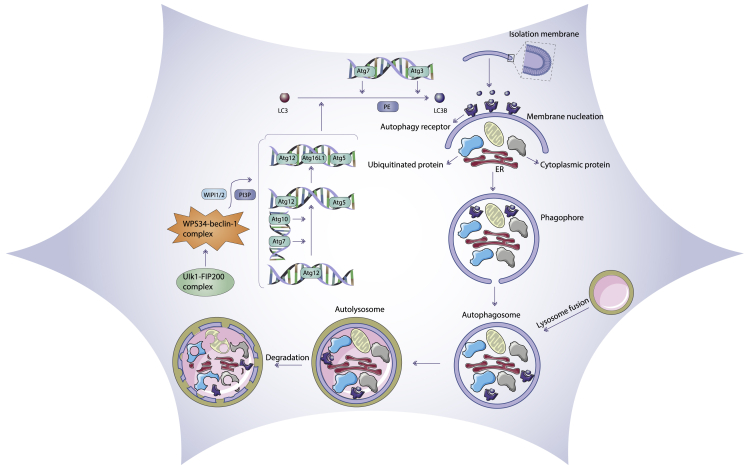
Mechanism of the autophagy pathway A series of stepwise processes, i.e., initiation, nucleation, elongation, maturation, and fusion with lysosomes, are involved in autophagy. Autophagy is induced at a basal level under normal conditions and is further stimulated by stress, for example, by nutrient deprivation

Autophagy is mainly a cytoprotective process designed to protect against starvation, in which cytoplasmic components undergo sequestration, autophagosomal transport to lysosomes, and subsequent degradation^33^, ^34^, ^35^ (Figure 4). It is notable that lysosomes have the ability to digest these components, which can then be recycled for the creation of new cellular proteins and new organelles or be additionally processed and used as a source of energy. Moreover, autophagy may be activated by diverse stresses, such as nutrient deprivation (caloric restriction), or may be caused by signals arising in the course of cellular differentiation or embryogenesis, and it can also be triggered by damaged intracellular organelles.^36^ In addition, a basal level of autophagy exists that results in the physiological turnover of damaged organelles, cytoplasmic contents, as well as longer-lived proteins.^37^

During the initial step of autophagy, a sensor called the mechanistic (previously mammalian) target of rapamycin (mTOR) is activated to form complex I (mTORCI), in response to several autophagy inducers. mTORCI can stimulate autophagy via initiating a signal transduction cascade by phosphorylating selected protein components of the autophagy machinery.^38^ mTORCI centrally contributes to the modulation of the ULK1-ATG13-FIP200 (FAK family-interacting protein of 200 kDa) complex, as shown by the inhibition of mTORCI with rapamycin increasing the kinase activity of ULK1.^39^ Researchers have divided phosphatidylinositol 3-kinase (PI3K) enzymes into three different isoforms (class I, II, and III). The mTOR signaling pathway is stimulated by class I PI3K, which also suppresses autophagy, whereas the class III PI3K isoform (Vps34) activates autophagy. There is not enough information about class II PI3K activity and autophagy.^40^ In addition, mitogen-activated protein kinase (MAPK) signaling may activate autophagy via activation of AMPK and consequent autophagy-associated gene translation. Therefore, PI3K-AKT signaling suppresses autophagy by activating mTOR and suppressing p53. Furthermore, the Vps34 complex combines with Beclin-1 to increase autophagic cell death. However, the binding of ATG5 to ATG12, as well as ATG16L, and phosphatidylethanolamine (PE) to the microtubule-related protein 1 light-chain 3 (LC3) is crucial to initiate and develop autophagy.^41^ Once initiated, the autophagy process will further progress to carry out its function.

Autophagy is designed either to enhance cell survival in physiologically stressed conditions, or to provoke an alternative programmed cell death pathway. In stressful conditions such as deprivation of growth factors or nutrients, autophagy ensures that cells receive enough vital metabolic substrates, thereby compensating for the metabolic stress imposed on the cells and enhancing cell survival.^42^, ^43^, ^44^, ^45^ Furthermore, autophagy has been found to be induced in cells that are detached from the extracellular matrix (ECM) in order to avoid anoikis cell death.^46^ While the main function of autophagy is the recycling of cellular contents, it can also play a role as a cell death pathway, especially to dispose of aging cells and neoplastic cells.^36^ Unlike apoptotic cell death, autophagy is a reversible mechanism, which can be reduced after elimination of the autophagy-stimulating stress.^47^ However, both apoptosis and autophagy can function either to regulate normal cell activities such as cellular homeostasis and physiological recycling of cell contents, or else to trigger cell death when necessary.

### Anoikis

Originating from a Greek word meaning homelessness, anoikis is another type of cell death induced by the detachment of adherent cells from the ECM (Figure 3).^48^ It has also been physiologically associated with tissue homeostasis, embryonic development, and the pathogenesis of diseases.^49^, ^50^, ^51^

Anoikis can be considered to be a subset of the apoptosis mechanisms,^52^, ^53^, ^54^ and it can also be categorized into intrinsic and extrinsic pathways.^55^ As mentioned earlier, the intrinsic pathway requires caspase activation due to permeabilization of the outer mitochondrial membrane via pro-apoptotic protein members of the Bcl-2 family.^56^ Likewise, the extrinsic pathway involves death receptors like TNF superfamily receptors, and ultimate activation of caspase-8. The two pathways have no biological distinction because the intrinsic pathway may be induced by caspase-8, which is also activated by the extrinsic pathway.^57^^,^^58^

The initiation of anoikis depends on proteins mediating the cellular interaction with the ECM and with neighboring cells, such as E-cadherin and integrins. Integrins are cell membrane receptors consisting of two components, α and β subunits. When integrins are ligated to their ECM receptors, they activate pro-survival signaling pathways, such as the PI3K/Akt pathway.^59^ Alternatively, integrins trigger anoikis and apoptosis when they adopt an unligated conformation.^58^ The apoptotic response of integrins is mediated by recruiting caspase-8.^60^^,^^61^ E-cadherin is a transmembrane protein with a cytoplasmic domain that is bound to β-catenin, which then binds to α-catenin. Because α-catenin binds to actin filaments, this complex mediates the binding E-cadherin to the cytoskeleton, and it regulates the adhesion between neighboring cells.^62^^,^^63^ In addition, E-cadherin can trigger anoikis via the RAF/extracellular signal-regulated kinase (ERK) and PI3K/Akt pathways.^64^^,^^65^ The loss of E-cadherin contributes to anoikis evasion in hepatocyte spheroids in the absence of ECM.^66^ Hence, the loss of E-cadherin and the gain of N-cadherin are the hallmarks of the epithelial-to-mesenchymal transition (EMT). The EMT is characterized by the loss of anoikis, and it allows detached cancer cells to survive long enough to produce metastasis.^67^^,^^68^ As mentioned above, the main factor responsible for initiating anoikis is cell detachment from the surrounding ECM. The appropriate interactions between adjoining cells and between cells and the surrounding matrix are a key factor for the survival and differentiation of epithelial cells. Regarded as a critical point in carcinogenesis,^69^^,^^70^ anchorage-independent growth results from decreased cell-ECM interactions, and in normal cells the resultant integrin signaling triggers anoikis.^55^ Alternatively, the appropriate anchorage between cells and matrix downregulates anoikis in normal cells.^49^ However, one characteristic of tumor cells is that they can circumvent anoikis when they become detached from their ECM. Therefore, cancer cells can easily invade the surrounding tissues after becoming detached from their primary location.^51^^,^^71^, ^72^, ^73^ Some virus-infected cells have been also proven to be resistant to anoikis-mediated cell death.^74^^,^^75^ For instance, anchorage-independent high-risk human papillomavirus (hrHPV)-transformed cells avoid anoikis, probably by inactivation of tumor suppressor genes.^76^

### miRNAs and cell death pathways

Recently, miRNAs have become popular targets for controlling and treating a variety of diseases. miRNAs have been applied in the clinical settings as diagnostic biomarkers for several diseases, and they are also being investigated in vaccine development.^7^

Although miRNAs are mostly considered to act as post-transcriptional regulators, their regulatory function has been also described at transcriptional levels. The effects of miRNAs on the target gene mRNA are largely dependent on the degree of complementarity between the target-gene transcriptional sequence and the sequence of the miRNA. There are two possible scenarios. (1) When the miRNA is fully complementary to the target gene mRNA, the function is the same as that of small interfering RNA (siRNA) and ultimately leads to cleavage of the target mRNA. (2) When the miRNA is not perfectly complementary to the target gene mRNA, it can still bind and thus inhibits translation by interfering with the mRNA stability.^77^

A wide variety of cellular processes have been reported to be regulated by miRNAs, the most important of which is cell death processes (apoptosis, autophagy, anoikis). This has been proposed to be a key mechanism for treating several serious conditions.

Defects in the regulation of apoptosis (either too little or too much) are involved in several human diseases, such as neurodegenerative conditions, ischemic tissue damage, autoimmune disorders, and in numerous types of cancer.^24^ Slattery et al.^22^ described the involvement of multiple miRNAs in the process of apoptosis, both in the intrinsic and extrinsic pathways through regulating the expression of specific genes (extrinsic pathway genes CASP7 and BIRC5, and intrinsic pathway genes CSF2RB and BCL2) in colorectal cancer. In another similar study, some apoptosis-associated genes, including *BCL2* and *PUMA* (a Bcl-2 family member), were found to be regulated by various miRNAs, such as miR-148a, miR-125b, miR-143, and miR-203.^78^, ^79^, ^80^, ^81^ Furthermore, miRNAs have been reported to regulate other targets in apoptosis-related pathways, such as PEG10, BTG1, ID1, interleukin (IL)-32, and NCF2.^82^

miRNAs can target several autophagy-related complexes, thereby modulating autophagy at various stages, including initiation, vesicle nucleation, elongation, and completion.^83^ miRNAs can regulate autophagy by targeting autophagy proteins such as beclin (the first autophagy protein to be discovered), or autophagy-related signaling pathways such as AMPK-mTOR.^41^ For example, miR-30a can inhibit autophagy by downregulating beclin expression, and miR-138 can upregulate the AMPK-mTOR pathway.^84^^,^^85^

Because autophagy and apoptosis share some similar intracellular pathways involved in cellular homeostasis, they also share some upstream mediators, such as specific miRNAs.^86^^,^^87^ Studies have confirmed the ability of certain miRNAs to inhibit both processes. For instance, there is a physical interaction between BECN1 and the anti-apoptotic family members (BCL2, MCL1, and BCL2L1), which is crucial for connecting the two pathways. Under normal conditions, BECN1 and anti-apoptotic BCL2 family members are capable of binding to each other to maintain cellular homeostasis. However, in situations where the cells are subjected to stressful conditions, the complex between BECN1 and the BCL2 family members disassociates, increasing autophagy and suppressing apoptosis.^88^, ^89^, ^90^ Furthermore, *MIR30A* is capable of reducing the cytoplasmic level of BECN1, while numerous other miRNAs can decrease the expression of anti-apoptotic family members, such as BCL2 and MCL1.^91^^,^^92^ Therefore, miRNAs can have an active involvement in the regulation of autophagy and apoptosis by altering protein-protein interactions. In addition, SQSTM1 (sequestosome-1) has been proposed to be another mediator that is controlled by miRNAs. One study showed the ability of SQSTM1 to modulate the polyubiquitination and aggregation of CASP8 that is crucial for the extrinsic apoptosis pathway.^93^ Put differently, SQSTM1 is capable of negatively regulating the degradation of autophagic protein LC3 via the 20S proteasome.^94^ The mechanism underlying the interaction between SQSTM1 and the apoptosis and autophagic pathways is being further investigated.

Various genes simultaneously take part in the autophagy and apoptosis pathways, including factors such as DAPK1 (death-associated protein kinase 1), EEF2 (eukaryotic translation elongation factor 2), and some signal transduction mediators such as *TP53* (*p53*) and *ATG5*.^86^^,^^95^^,^^96^ Some miRNAs can exert a regulatory effect on common targets in both apoptosis and autophagy pathways, thus regulating both of them at the same time. *MIR101*, for instance, regulates a gene shared between the two pathways, which in turn controls the expression of RAB5A, STMN1, and ATG4D as autophagic proteins and MCL1 as an anti-apoptotic protein.^97^^,^^98^ During hypoxia-reoxygenation endogenous *MIR204* promoted the effect of chemotherapy to induce apoptosis via BCL2 downregulation, but it inhibited autophagy through LC3-II protein modulation.^99^^,^^100^

Besides apoptosis and autophagy, anoikis cell death is also affected by various miRNAs. For instance, hsa-miR-34c-3p/5p,^101^ hsa-miR-203,^102^ and hsa-mir-129-2/-137/-3663^103^ were all shown to be involved in modulating anchorage-independent growth and therefore anoikis. It was suggested that methylation-dependent downregulation of tumor suppressive miRNAs played a major role in anchorage-independent growth and in circumventing anoikis.^76^^,^^103^ Once the cancer cells or the virus-infected cells are fully anchorage-independent, they may bypass anoikis and further spread throughout the body, thereby disseminating the disease. Moreover, several miRNAs have been recently found to be directly associated with either the positive or negative regulation of anoikis.^104^ miRNA 204,^105^, ^106^, ^107^, ^108^ miR-200c,^109^, ^110^, ^111^ and miR-146a^112^, ^113^, ^114^, ^115^ can regulate anoikis in cancer cells, whereas some other miRNAs such as miR-107^75^ and miR-30a^116^ can perform a similar function in virus-infected cells. Hence, a broad spectrum of cellular mechanisms, especially cell death pathways, have been reported to be regulated by miRNAs in recent decades. In this review, we summarize the cell death-regulatory functions of miRNAs that can be dysregulated in virus infections (Table 1).

**Table 1:** Virus-regulated miRNAs known to play roles in modulating cell death pathways

Virus	Viral protein	miRNA	Expression of miRNA	Target of miRNA	Inhibition/ induction of cell death	Disease	Sample type	Ref.
HPV 16	E7	miR-27b	up	polo-like kinase2	inhibition of apoptosis	cervical cancer	*in vitro* (SiHa and CaSki)	^31^
HPV 16	E7	miR-21	up	–	inhibition of apoptosis	cervical cancer	*in vitro* (HeLa)	^117^
HPV 16 and 18	E6	miR-34a	down	–	inhibition of apoptosis	cervical cancer	human (cervical cancer tissue samples) *in vitro* (SiHa, HeLa, CaSki)	^118^
HPV 16	E6	miR-2861	down	CCND1, EGFR, AKT2	inhibition of apoptosis	cervical cancer	human (n = 57 cervical cancer tissue samples) *in vitro* (SiHa and CaSki)	^119^
HPV 16	E6	miR-23b	down	c-MET	inhibition of apoptosis	cervical cancer	*in vitro* (SiHa)	^120^
HPV 16	E5	miR-196a	down	HoxB8	inhibition of apoptosis	cervical cancer	*in vitro* (HeLa, SiHa)	^121^
HPV 16 and 18	E6, E7	miR-18a	up	STK4	inhibition of apoptosis	cervical cancer	human (cervical cancer tissue samples) *in vitro* (SiHa, HeLa, CaSki, SW756, C33A)	^122^
EBV	EBNA2	miR-34a	down	PD-L1	inhibition of apoptosis	Burkitt’s lymphoma	*in vitro* (Mutu I and Mutu III, Daudi, Jijoye, LCL, OMA4, U2932)	^123^
EBV	LMP2A	miR-155-5p	up	Smad2	inhibition of apoptosis	gastric cancer	*in vitro* (GT39, SNU719, EBVnGC, HGC27, SGC7901)	^124^
EBV	LMP1	miR-21	up	PDCD4, Fas-L	inhibition of apoptosis	nasopharyngeal carcinoma	*in vitro* (C666-1 HONE1, CNE2)	^125^
EBV	LMP1	miR-155	up	UBQLN1	inhibition of apoptosis	nasopharyngeal carcinoma	human (n = 8 radio-resistant tissue samples of NPC) *in vitro* (CNE-1, CNE-2)	^126^
EBV	–	miR-155	up	–	inhibition of apoptosis	B cell lymphoma	*in vitro* (EF3D, SDLCL)	^32^
EBV	LMP1	miR-155	up	PTEN	inhibition of apoptosis	B cell lymphoma	*in vitro* (SKW6.4 rituximab resistance)	^127^
EBV	EBNA1	miR34a	down	–	inhibition of apoptosis	gastric cancer	*in vitro* (SNU719)	^128^
EBV	–	miR-194	down	IL-10	inhibition of apoptosis	PTLD	human (n = 6 PBMC samples of PTLD) *in vitro*	^129^
HBV	HBx	miR-21	up	IL-12	inhibition of apoptosis	HCC	*in vitro* (HepG2.2.15)	^130^
HBV	HBx	miR-21	up	PDCD4, PTEN	inhibition of apoptosis	HCC	*in vitro* (HepG2, Huh7)	^131^
HBV	HBx	miR-181a	up	PTEN	inhibition of apoptosis	HCC	*in vitro* (HepG2)	^132^
HBV	HBx	miR-192-5p	up	BIM	inhibition of apoptosis	HCC	*in vitro* (HepG2)	^133^
HBV	HBx	miR-331-3p	up	ING5	inhibition of apoptosis	HCC	human (HCC tissue samples) *in vitro* (HepG2.2.15, SMMC7721)	^134^
HBV	HBx	miR-602	up	RASSF1A	inhibition of apoptosis	HCC	human (n = 21 HCC tissue samples) (HepG2.2.15, HepG2-HBX)	^135^
HBV	–	miR-15a	down	Smad7	inhibition of apoptosis	HCC	human (n = 40 HCC tissue samples) *in vitro* (HepG2-4D14, HepG2-C5)/*in vivo*	^136^
HBV	–	miR-30e-5p	down	MAP4K4	inhibition of apoptosis	HCC	human (n = 55 HCC tissue samples) *in vitro* (HepG2.2.15, Hep3B)	^137^
HBV	–	miR-15a/16 Cluster	down	Bcl-2	inhibition of apoptosis	HCC	human (n = 40 HCC tissue samples) *in vitro* (HepG2.2.15)/*in vivo*	^138^
HBV	HBx	miR-1236	down	alpha-fetoprotein (AFP)	inhibition of apoptosis	HCC	human (n = 97 tissue samples of HCC) *in vitro*/*in vivo*	^139^
HBV	HBx	miR-1270	down	alpha-fetoprotein (AFP)	inhibition of apoptosis	HCC	human (n = 97 HCC tissue samples) *in vitro*/*in vivo*	^139^
HBV	–	miR-101-3p	down	Rab5a	inhibition of apoptosis	HCC	*in vitro* (SMMC-7721, HepG2.2.15)	^140^
HBV	HBx	miR-15a and 15b	down	CCND1	inhibition of apoptosis	HCC	*in vitro* (SK-HEP-1, Huh7, HepG2)	^141^
HBV	HBx	miR-16	down	CCND1	inhibition of apoptosis	HCC	*in vitro* (SK-HEP-1, Huh7, HepG2)	^141^
HBV	HBx	let-7	down	–	inhibition of apoptosis	HCC	human (n = 19 HCC tissue samples) *in vitro* (HepG2.2.15)	^142^
HBV	HBx	miR-193b	down	–	inhibition of apoptosis	HCC	human (HCC tissue samples) *in vitro*	^143^
HBV	HBx	miR-548p	down	HBXIP	inhibition of apoptosis	HCC	human (n = 21 HCC tissue samples) *in vitro* (Hep3B, HepG2.2.15)	^144^
HBV	HBx	miR-375	down	AEG-1	inhibition of apoptosis	HCC	*in vitro* (SMMC-7721, HepG2-HBx)	^145^
HBV	HBx	miR-136	down	AEG-1	inhibition of apoptosis	HCC	*in vitro* (SMMC-7721, HepG2-HBx	^145^
HBV	HBx	miR-192	down	–	inhibition of apoptosis	HCC	*in vitro* (HepG2)	^146^
HBV	HBx	miRNA-145	down	CUL5	inhibition of apoptosis	HCC	human (n = 25 HCC tissue samples) *in vitro* (HepG2.2.15)	^147^
HBV	HBc, HBx	miR-328-3p	up	FOXO4	inhibition of apoptosis	–	*in vitro* (THLE-2)	^148^
HBV	–	miR-98-5p	up	NF-κB	induction of apoptosis	HCC	human (n = 30 HCC tissue samples) *in vitro* (MHCC97H, Huh7)	^149^
HCV	–	miR-155	up	APC	inhibition of apoptosis	HCC	human (n = 10 HCC tissue samples) *in vitro* (Huh7)/*In vivo*	^150^
HCV	core	miR-345	up	p21^Waf1/Ci1^	inhibition of apoptosis	–	*in vitro* (Huh7)	^151^
HCV	core	miR-93	up	–	inhibition of apoptosis	–	*in vitro* (Huh7)	^151^
HCV	core	miR-30c	down	–	inhibition of apoptosis	HCC	human (n = 152 HCC tissue samples) *in vitro* (HepG2)	^152^
HCV	core	miR-203a	down	–	inhibition of apoptosis	HCC	human (n = 152 HCC tissue samples) *in vitro* (HepG2)	^152^
HCV	NS5A	miR-503	down	Bcl-2	inhibition of apoptosis	–	*in vitro* (HepG2)	^153^
HCV	–	miR-193b	down	Mcl-1	inhibition of apoptosis	HCC	*in vitro* (Hep-394)	^154^
HCV	–	miR-181c	down	ATM	inhibition of apoptosis	–	human (n = 8 tissue samples of chronically HCV-infected) *in vivo* (HepG2)	^155^
HTLV-1	Tax	miR-155	up	–	inhibition of apoptosis	ATLL	*in vitro* (MT-2, C5/MJ, ED-40515)	^156^
HTLV-1	Tax	miR-130b	up	TP53INP1	inhibition of apoptosis	ATLL	human (PBMC samples of ATLL) *in vitro* (MT-4)	^157^
HTLV-1	–	miR-93	up	TP53INP1	inhibition of apoptosis	ATLL	human (PBMC samples of ATLL) *in vitro* (MT-4)	^157^
Influenza A	–	miR-29c	up	BCL2L2	induction of apoptosis	influenza	*in vitro* (A549)	^158^
GaHV-2	Meq	miR-21	up	PDCD4	inhibition of apoptosis	Marek’s disease, lymphoma	*in vitro* (MSB-1)	^159^
Rotavirus	NSP5	miR-142-5p	up	SMAD3, TGFβR2	inhibition of apoptosis	–	*in vitro* (HT29)	^160^
HSV-1	–	miR-23a	up	IRF1	inhibition of apoptosis	–	*in vitro* (HeLa)	^161^
HBV	HBx	miR-194-5p	up	cFLIP, SODD	induction of apoptosis	HCC	*in vitro* (HepG2)	^133^
HCV	–	miR-200c	up	FAP1	induction of apoptosis	hepatic fibrosis	human (n = 10 tissue samples of HCV fibrosis)	^162^
Influenza A (H1N1)	–	miR-34a	down	Bax	induction of apoptosis	influenza	human (n = 10 influenza serum samples) *in vitro* (A549)	^163^
Enterovirus 71	–	let-7b	up	CCND1	induction of apoptosis	–	*in vitro* (SH-SY5Y)	^164^
West Nile virus	–	Hs_154	up	ECOP, CTCF	induction of apoptosis	–	*In vitro* (SK-N-MC and HEK293)	^165^
RSV	NS1	miR-24	down	–	induction of apoptosis	–	*In vitro* (A549)	^166^
HBV	HBx	miR-192-3p	down	XIAP	induction of autophagy	hepatitis B	human (serum samples of hepatitis B) *in vitro* (HepG2.2.15)	^167^
HBV	HBsAg	miR-155	up	SOCS1/Akt/mTOR	induction of autophagy	–	*in vitro* (HepG2, HepG2.2.15)	^168^
Enterovirus 71	–	miR-30a	down	Beclin-1	induction of autophagy	–	*in vitro* (Vero, Hep2)	^116^
HBV	–	miR-224	up	Smad4	inhibition of autophagy	HCC	human (HCC tissue samples) *in vivo*	^169^
HPV 16	E7	miR-106a	up	LKB1	inhibition of autophagy	cervical cancer	human (n = 91 cervical cancer tissue samples) *in vitro* (SiHa, HeLa, CaSki, HEK293T)	^170^
HBV	HBx	miR-7	up	maspin	inhibition of anoikis	HCC	human (n = 69 HCC tissue samples) *in vitro* (HepG2x, Hep3Bx, HepG2.2.15)	^75^
HBV	HBx	miR-107	up	maspin	inhibition of anoikis	HCC	human (n = 69 HCC tissue samples) *in vitro* (HepG2x, Hep3Bx, HepG2)	^75^
HBV	HBx	miR-21	up	maspin	inhibition of anoikis	HCC	human (n = 69 HCC tissue samples) *in vitro* (HepG2x, Hep3Bx, HepG2)	^75^

PBMC, peripheral blood mononuclear cell.

### RNA-binding proteins in virus-miRNA interactions

RNA-binding proteins are involved in the function of miRNAs in various conditions such as viral infections. For example, flaviviruses, which are single-stranded (ss)-positive RNA viruses, interfere with the miRNA processing machinery by allowing the accumulation of the non-coding (nc) subgenomic flavivirus RNA (sfRNA) that then carries out sequestration of the double-stranded RNA (dsRNA) binding proteins Dicer and AGO2.^171^ Dicer interacts with the transactivation response RNA-binding protein (TRBP) and the protein activator of protein kinase R (PKR) (PACT), two dsRNA-binding proteins that play roles in the efficiency and specificity of miRNA processing.^172^

Alternatively, some miRNAs such as miR-101 also downregulate the RNA-binding protein G-rich sequence factor 1 (GRSF1), whose binding to herpes simplex virus 1 (HSV-1) p40 mRNA typically increases its expression, facilitating viral replication. Therefore, induction of miR-101 expression during HSV-1 infection downregulates both ATP5B and GRSF1 expression, consequently attenuating viral replication and preventing lytic cell death.^173^

It was reported that PCBP2, a member of the heterogeneous nuclear ribonucleoprotein (hnRNP) E family of RNA-binding proteins, can interact with a sequence-specific motif of single-stranded poly(C) polynucleotides and is involved in the regulation of defense responses to viruses and the negative regulation of immune effector cells. PCBP2 could serve as a target gene of miR-HA-3p in H5N1 influenza virus-induced uncontrolled immune responses. In addition to regulating mRNA translation and maintaining mRNA stability, PCBP2 is essential for the prevention of excessive immune responses by serving as a pivotal negative regulator in the mitochondrial antiviral signaling protein (MAVS) signaling pathway.^174^

Furthermore, the miR-17/92 cluster and miR-20a have been reported to target the p300-cyclic AMP (cAMP)-responsive element binding protein (CREB)-binding protein-associated factor (PCAF) and render the cells less susceptible to HIV-1 infection. It is known that PCAF has a role in tat acetylation, and acetylated tat is transcriptionally more active.^81^ Alternatively, HIV-1 tends to suppress these miRNAs and upregulate PCAF expression, leading to a possible increase in viral infectivity.^175^^,^^176^

Viruses have had ample time to evolve in ways that prevent the binding of RNA-induced silencing complexes (RISCs) programmed by cellular miRNAs, leading to their destruction. The rapid loss of cognate RISC-binding sites has been observed when rapidly evolving viruses such as HIV-1 are grown in the presence of artificial siRNAs, which function in a similar manner to miRNAs when they bind to perfectly complementary RNA targets.^177^^,^^178^ Moreover, viral gene products that block RISC function are common in plants and insect viruses, where RNAi represents a major innate immune response to viral infection. It remains unclear whether viruses that infect mammals, which do not mount an innate RNAi response to viral infection of somatic cells, commonly encode inhibitors of miRNA biogenesis.^179^

### Therapeutic potential of miRNAs in virus infections

miRNAs are involved in the regulation of almost all physiological processes, such as cell development, differentiation, proliferation, and apoptosis. In this context, miRNAs may have great potential as new biomarkers for diagnosis and in therapeutic approaches for viral infections. To date, more than 1,000 different human miRNAs have been identified, and it is predicted that miRNAs control the expression of approximately 60% of all human genes.^180^ Recently, new methods and tools have been developed for the detection and quantification of virus-regulated miRNAs. For example, the use of multiplexed qRT-PCR, microarrays, or next-generation sequencing (NGS)-based genome-wide approaches have helped to provide expression profiles of large numbers of miRNAs.^181^ Moreover, bioinformatics approaches and existing databases have enabled the discovery of thousands of miRNAs in viruses, e.g., Vir-Mir db, BiTargeting, and ViTa.^182^

The aberrant expression of miRNAs has been linked to the initiation and progression of virus infections. Some miRNAs have been demonstrated to be involved in the pathogenesis of viral diseases, e.g., mir-122 and mir-21 in hepatitis C virus (HCV), and mir-507 and mir-136 in influenza.^183^

Furthermore, the structure and quantity of miRNAs contained within exosomes secreted from virally infected and non-infected cells are different.^184^ Therefore, miRNAs could provide a new approach for better clinical decision-making. Moreover, miRNAs carry out potent and specific gene silencing, making them attractive therapeutic tools. To date, most efforts in this setting have been to explore the potential application of miRNA therapeutics for viral infections.^185^

However, some challenges faced by the use of miRNAs as therapeutic tools include: (1) degradation by nucleases after introduction into biological systems, (2) poor cell membrane penetration, (3) entrapment in endosomes, (4) insufficient binding affinity for complementary sequences, (5) inadequate delivery to desired target tissues, (6) off-target effects and unwanted toxicity, and (7) activation of innate immune responses.^186^ Despite these limitations, future research could improve the miRNA properties to overcome these challenges. For example, improvements in miRNA delivery are being addressed by chemical modifications and applications of nanotechnology.^186^

## Indirect regulation of the apoptosis pathway in viral infections by cellular miRNAs

A vast array of virus-related miRNAs have been found to be involved in the modulation of cellular apoptosis.

### HBV

Several miRNAs are known to participate in the life cycle of HBV infections.^187^ HBV causes acute and chronic hepatitis in humans, and it has become a serious health issue affecting 240 million individuals worldwide.^188^^,^^189^ Epigenetic modifications occurring in HBV infection increase the expression of miR-1 and miR-449a and in turn increase the expression of the nuclear receptor farnesoid X receptor α (FXRα), which stimulates the core HBV promoter function as well as HBV replication.^190^^,^^191^ Moreover, miR-373 and miR-372 are capable of upregulating HBV gene expression via the nuclear factor (NF)-κI/B axis.^192^ It was found that the miR-122 levels were negatively correlated with intrahepatic viral load, hepatic necrosis, and inflammation. The depletion of endogenous miR-122 by its antisense inhibitor led to enhanced HBV replication, whereas overexpression of miR-122 by transfection using a mimic or an expression vector inhibited viral production. miR-122 can have an indirect effect on HBV replication via repression of the expression of its target cyclin G1, thereby inhibiting the binding of p53 to cyclin G1 and abrogating the p53-regulated suppression of HBV replication. Therefore, miR-122 can act as a host restriction factor in HBV infection, and its downregulation during HBV infection might contribute to viral persistence. Therefore, the induction and increase of miR-122 expression could provide an effective strategy for treatment of HBV infection and related liver diseases.^192^ In contrast, miR141 could downregulate HBV replication by targeting the peroxisome proliferator-stimulated receptor-α (PPARA). Hence, miR-141 could suppress HBV replication by reducing HBV promoter activity by downregulating PPARA. PPARA may be a promising host-oriented drug target for the development of novel HBV treatment, which could be mediated by miRNAs.^193^ miR-130a targets two metabolic modulators, peroxisome proliferator-stimulated receptor-γ and its respective co-activator PGC1-α, thereby preventing replication of HBV.^194^ miRNAs are important regulators in viral infections, which could provide an effective strategy for treatment of HBV infection and related diseases.

Aquaporins (AQPs) are membrane water transport channels that contribute to water secretion and adsorption in epithelial cells. A number of aquaporin subtypes can transfer other molecules, such as urea and glycerol. At present, there are 13 specific types of AQPs in mammals that are principally divided into three groups:^195^^,^^196^ (1) classical AQPs that are the original water-selective channels, including AQP0, AQP1, AQP2, AQP4, AQP5, AQP6, and AQP8; (2) aquaglyceroporins such as AQP3, AQP7, AQP9, and AQP10 that are capable of transporting glycerol and other small solutes; and (3) non-classical AQPs such as AQP11 and AQP12 that are located inside the cells. Nonetheless, researchers have not yet reached a complete understanding of their selectivity. Currently, the contribution of the AQPs to tumor progression is increasingly being investigated. Earlier investigations showed strong expression of the AQPs in tumor cells from various origins that were proposed to contribute to tumor-related edema, tumor cell migration, proliferation of tumors, as well as tumor angiogenesis.^197^^,^^198^ In addition, the expression of AQPs in various types of tumors is different due to the respective tissue-specific localization. Furthermore, the expression of AQP5 is upregulated in colon cancer tissues.

Zhang et al.^199^ addressed the role of miR-325-3p and AQP5 in the proliferation and apoptosis of HBV-infected hepatocellular carcinoma (HCC) cells. The results showed that miR-325-3p directly targeted AQP5 and decreased mRNA and the protein level of AQP5 that increased proliferation and inhibited apoptosis in HCC cells. Overexpression of miR-325-3p further inhibited proliferation and induced apoptosis. Introducing miR-325-3p into the cells inhibited growth and induced apoptosis in Huh7-1.3 and HepG2.2.15 cells via a direct decrease of AQP5 expression. Moreover, silencing the AQP5 expression duplicated the pro-apoptotic impact of miR-325-3p overexpression. HBV downregulates miR-325-3p and suppresses AQP5, resulting in inhibition of apoptosis and contributing to tumor progression.^199^ Therefore, AQPs may be a promising drug target for the development of a novel HBV therapy that uses miRNAs.

### Avian leukosis viruses (ALVs)

ALVs are a type of avian retroviruses that can induce tumors in chickens.^200^ Chicken ALVs are categorized into six subgroups (A–E and J) with regard to the envelope glycoprotein that is responsible for the viral entry and is involved in host virus neutralization.^201^^,^^202^ During recent years, ALV-J has become epidemic in nature and affects commercial egg layers and local breeding flocks in China.^203^ Moreover, ALV-J causes myeloid leukosis and creates more severe problems in comparison to other virus subgroups.^204^ Studies have shown that ALV-J and other subgroups are among the leading causes of avian infections^205^, ^206^, ^207^ and can also cause immunosuppression by inhibiting dendritic cells (DCs).^208^ DCs are a type of professional antigen-presenting cells (APCs) that have multiple subtypes with different functions. DCs are capable of promoting an immune response in the presence of tissue damage signals or pathogens, and also inducing and maintaining immune tolerance.^209^ Moreover, DCs have been proposed to be the master regulators and initiators of the immune responses that link innate and adaptive immunity.^210^^,^^211^ In addition, their existence is crucial for maintaining immune homeostasis and preventing autoimmune responses to self-antigens.^210^ However, their function relies on suitable DC maturation that is often modulated by miRNAs.^212^, ^213^, ^214^, ^215^

The ALV provirus, as a retrovirus, is capable of random integration into the host genome that possibly leads to the deregulation of gene expression, in particular the expression of diverse regulatory factors such as miRNAs.^216^ Moreover, there is insufficient information about the mechanism of the ALV provirus integration, and ALV integration is frequently random. Nonetheless, a number of investigations have shown the integration of the provirus into specific sites upstream of some oncogenes (e.g., c-erb, c-*myc*, TERT, EGFR, MET, ZIC1).^217^^,^^218^ Therefore, ALV integration may modulate the expression of some miRNAs by unclear mechanisms. In addition, the process of virus infection, as well as replication, triggers the cells to alter their miRNA expression. Because some miRNAs, such as miR-146a and miR-146b, are responsible for DC apoptosis,^219^ the immunosuppressive effect of ALV-J on DCs may be attributed to the regulation of miRNAs involved in apoptosis pathways.

Liu et al.^220^ explored the miRNA-mediated apoptotic effects of ALV infection on cultured DCs *in vitro*. Based on their findings, ALV-J was shown to be capable of infecting the cells in the initial phases of differentiation, and infection with ALV-J caused apoptosis in the DCs. Moreover, miRNA sequencing of the non-infected and infected DCs showed 122 differentially expressed miRNAs, of which 115 were upregulated following ALV-J infection, and 7 miRNAs showed considerable downregulation. The miRNAs exhibiting the most upregulation were proposed to affect nutrient processing as well as metabolic functions. Therefore, ALV-J infection of the chicken DCs was capable of inducing apoptosis through altered miRNA expression, providing the motivation for additional study of the epigenetic effects of ALV-J-induced immunosuppression. Therefore, aberrant miRNA expression caused by ALV-J infection may interfere with DC differentiation and apoptosis, resulting in defective antigen presentation and suppression of the immune response.^220^

### Chikungunya virus (CHIKV)

CHIKV is a re-emerging infectious disease caused by an alphavirus, resulting in large-scale outbreaks in the islands of the Indian Ocean, Southeast Asia, and currently in the Caribbean region.^221^, ^222^, ^223^ Moreover, CHIKV causes symptoms of debilitating arthritis that can endure from 1 week up to a year.^224^^,^^225^ CHIKV can infect both peripheral tissue as well as the central nervous system (CNS), which is most serious in newborns and children, and even in adults.^225^ For peripheral infections, CHIKV virus can affect the connective tissue of the joints, fibroblast cells of the dermis, as well as the muscle fascia. In addition, CHIKV induces delayed apoptosis in infected cells, and it uses the apoptotic blebs for spreading the infection to the neighboring cells.^226^, ^227^, ^228^ However, further studies are needed to fully investigate the CHIKV-mediated molecular pathways in fibroblasts.^222^^,^^224^

Sharma et al.^229^ carried out an investigation on the regulation of miRNAs in mouse fibroblast cells immediately following infection in order to identify the pathways targeted by CHIKV. In the next step, the researchers analyzed the expression of 760 separate miRNAs at 6 h after infection with CHIKV. Bioinformatic analysis was used to identify the signaling pathways that were targeted by the highly altered miRNAs. A singleplex miRNA assay was used to validate the miRNAs, and then western blot analysis was used to confirm the protein targets of the miRNAs. Among the 760 miRNAs studied, it was found that 75 miRNAs were significantly regulated following CHIKV infection, and of these 71 were upregulated in the infected cells. According to the functional pathway and network analysis, the miRNAs that were most altered contributed to apoptosis and signaling pathways, such as Toll-like receptors (TLRs), the PI3K/Akt pathway, mTOR, and JAK-STAT, that were proposed to be involved in the course of CHIKV infection. Finally, topoisomerase Iiβ was one target of the two downregulated miRNAs in CHIKV infection. Several miRNAs have been shown to be involved in processes that occur later after CHIKV infection, such as cellular proliferation, immune response, and apoptosis, which may be useful for treatment of CHIKV infections in the future.^229^

### Influenza A virus (IAV)

IAV is one of the most common enveloped negative strand RNA viruses in the Orthomyxoviridae family. The IAV genome contains eight RNA sequences, with two of them encoding major surface glycoproteins known as neuraminidase (NA) and hemagglutinin (HA). By analysis of its antigenicity, this virus may be divided into 18 HA subtypes (H1–H18) and 11 NA subtypes (N1–N11). However, researchers have found a large number of permutations containing various HA and NA subtypes.^230^ Based on the virus strain, different IAVs have different pathogenicity for infection. For instance, less pathogenic avian influenza viruses (LPAIVs) result in only mild respiratory symptoms that can be occasionally followed by a gastrointestinal infection. Alternatively, the highly pathogenic avian influenza viruses (HPAIVs) such as H7N9 and H5N1 lead to multiorgan systemic infections, as well as fatal illnesses.^231^ Overall, IAV infections annually account for nearly 500,000 deaths throughout the world, and they also cause high rates of death in several animal species.^232^ The key clinical characteristic is the severity of pneumonia that manifests as cough, fever, and difficulties in breathing. Even though some investigations have shown that IAV can affect cell apoptosis *in vitro* and *in vivo*,^158^^,^^233^^,^^234^ there is not enough information about the respective molecular mechanism(s).

IAV infection is capable of altering miRNA expression in animal models as well as in cultured cells.^235^, ^236^, ^237^, ^238^ It is known that specific miRNAs are capable of positively or negatively influencing the immune response,^239^, ^240^, ^241^, ^242^ and some individual miRNAs can control apoptosis induced by virus infection by targeting cellular mRNAs and protein levels.^158^ Furthermore, changes in miRNA expression following viral infection profoundly influence the innate host response to viral infection. Nevertheless, the effects of miRNAs on cell apoptosis after IAV infection have been less well studied.

In one study, Fan and Wang^243^ addressed the molecular mechanism of miR-34a in IAV-induced apoptosis. They used a miRNA array to identify the miRNAs involved in IAV infection, and western blotting and a luciferase reporter assay to determine the target gene of miR-34a. After that, they transfected a miR-34a mimic into the IAV-infected A549 cells and examined the changes in target gene expression. According to the results, miR-34a was considerably downregulated in IAV-infected A549 cells, as confirmed by quantitative reverse transcriptase PCR (qRT-PCR) *in vivo* and *in vitro*. Afterward, transfection with a miR-34 mimic or an inhibitor in the IAV-infected A549 cells confirmed the effects on apoptosis. It was predicted that miR-34a was complementary to the 3′ UTR of the mRNA for Bax, and western blotting and a luciferase reporter assay confirmed the direct targeting of Bax by miR-34a. Western blotting showed that the upregulation of the Bax protein level was reversed by miR-34a overexpression. They proposed that miRNAs could provide therapeutic targets for influenza virus infections.^243^

## Indirect regulation of the autophagy pathway by cellular miRNAs in viral infections

It has been found that autophagy contributes to the interactions between host cells and viruses. In the course of viral infection, autophagy is capable of performing either a proviral or an antiviral function. Autophagy can act as an anti-viral defense mechanism of the innate immune system, because it can lead to the preferential degradation of the sequestered viral structures within the cells to inhibit virus replication.^244^, ^245^, ^246^ It has been proposed that several viruses, such as poliovirus, coronaviruses, enteroviruses, DENV, and HCV, can all subvert the autophagy mechanism to promote their respective replication.^116^^,^^247^, ^248^, ^249^, ^250^ Autophagy has been found to have a role in the regulation of virus replication, and it also directly affects the generation of some inflammatory cytokines. Disrupting the normal autophagy pathways considerably increases the secretion of the proinflammatory cytokines IL-1β, IL-18, and IL-1α.^251^, ^252^, ^253^ The treatment of human and murine cells with the autophagy inhibitor 3-methyladenine (3-MA) largely suppressed the TLR-dependent secretion of IL-6 and TNF-α.^254^ Moreover, silencing of ATG7 decreased the generation of TLR-dependent IL-8 in intestinal epithelial cells.^255^ Autophagy also contributes to the regulation of type I IFN secretion. Recently it was shown that knockdown of either ATG7 or Beclin-1 increased the expression of IFN-α and IFN-β in HCV infected immortalized human hepatocytes (IHHs).^256^ The innate immune system is affected by autophagy, and how this affects the virus-host cell interaction requires further investigation. Herein, we summarize the existing knowledge about the miRNAs that regulate autophagy in virus-infected cells.

### HBV and autophagy

Intracellular pathways, inflammatory cytokines, specific hormones, and other factors are known to be involved in the regulation HBV replication.^257^, ^258^, ^259^, ^260^ miRNAs have been found to be involved in the intracellular pathways modulating HBV transcription through regulation of transcription factors, nuclear receptors, and other gene products.^261^ miR-101 was found to repress the HBV replication via targeting the forkhead box O1 (FOXO1) transcription factor.^262^ miR-449a accelerated HBV replication via targeting CREB5, which in turn induced the expression of FXRα, a transcription factor known to facilitate HBV replication. CREB5 knockdown and overexpression confirmed that it was a negative regulator of HBV replication. Additionally, miR-449a overexpression inhibited proliferation, caused cell cycle arrest, and promoted HCC cell differentiation. The results indicated that epigenetically regulated miR-449a targeted CREB5 to increase FXRα expression, thereby promoting HBV replication and gene expression.^190^ Moreover, miR-224, miR-138, miR-224, miR-576, and miR-596 directly targeted the pre-genomic RNA (pgRNA) of HBV and inhibited virus replication. It has been proposed that in the early phase of HBV infection, HBx binding at or near to the p65/NF-κB sites within the miR-224 promoter leads to the repression of miR-224 expression and inhibits viral replication. However, inactivation of HBx leaves the miR-224 promoter free to respond to the TNF/LT-NF-κB signaling in HCCs, leading to the formation of highly dysplastic nodules.^263^ Nevertheless, this hypothesis remains to be confirmed *in vivo*.

The PI3K/Akt signaling pathway has been proposed to be a key cellular pathway in the regulation of HBV infection.^264^^,^^265^ In addition to the effects of apoptosis discussed in the previous section, autophagy is also involved in the regulation of HBV infection.^266^^,^^267^ IAV, HIV, HCV, and DENV can all take advantage of autophagy to enhance their replication.^250^^,^^268^, ^269^, ^270^, ^271^ Autophagy is thought to be modulated by the PI3K/Akt/mTOR pathway, which could facilitate HBV infection. For instance, autophagy was found to be increased by suppressing mTOR activity in eukaryotic cells.^272^^,^^273^

Zhang et al.^274^ investigated the role of miR-155 in HBV replication as well as the possible involvement of autophagy in this process. Moreover, downregulation of miR-155 increased HBV infection, while miR-155 transfection increased antigen expression, HBV replication, and progeny secretion in HepG2.2.15 cells. Additionally, miR-155 contributed to the suppressor of the cytokine signaling 1 (SOCS1)/Akt/mTOR axis and reinforced autophagy in HepG2.2.15 cells. Additionally, the autophagy inhibitor (3-MA) abolished the hepatitis B surface antigen (HBsAg) secretion triggered by miR-155. Hence, miR-155 helped HBV replication via reinforcing SOCS1-stimulated autophagy. It was proposed that chronic HBV could be treated or ameliorated by inhibiting miR-155 in the cells, and autophagy inhibitors (3-MA) could also be tested.^274^

Lin et al.^275^ examined whether the miR-99 family modulated HBV replication and investigated the underlying molecular mechanism. The expression of the miR-99 family was downregulated in hepatoma cells, in comparison to primary hepatocytes. Transfection of miR-99b, miR-100, and miR-99a enhanced HBV replication, antigen expression, and progeny secretion in hepatoma cells. Nonetheless, the miR-99 family did not influence HBV transcription or the activity of the HBV promoter, suggesting that these miRNAs modulated HBV replication at the post-transcriptional stage. In agreement with bioinformatic analysis, it was found that ectopic expression of the miR-99 family reduced the insulin growth factor (IGF)-1R/Akt/mTOR pathway signaling and suppressed insulin-stimulated activation in hepatoma cells. The miR-99 family increased autophagy via mTOR/ULK1 signaling and increased HBV replication.

Several miR-29 members, such as miR-29a, miR-29b, miR-29c, miR-29d, and miR-29e, have shown an anti-viral activity. Overexpression of miR-29c in HepG2.2.15 cells efficiently repressed the expression of TNF-α-induced protein 3 and inhibited HBV DNA replication. miR-29c overexpression suppressed proliferation and induced apoptosis.^276^ HCV infection downregulated miR-29, and the overexpression of miR-29 decreased the level of HCV RNA in hepatic stellate cells. HCV infection downregulates miR-29 in hepatocytes, and this may explain the increase in collagen synthesis by reducing the miR-29 levels in activated hematopoietic stem cells (HSCs). It is possible that treatment with a miR-29 mimic *in vivo* may suppress HCV and decrease fibrosis.^277^ Many studies have shown a correlation between the occurrence of fibrosis in different organs such as kidney,^278^ heart,^279^ and liver^280^ with members of the miR-29 family.

### Duck enteritis virus (DEV) and autophagy

DEV is the causative pathogen of duck viral enteritis disease that causes significant economic losses in the duck farming industry, resulting in the death of the birds and in lower egg production.^281^^,^^282^ Additionally, DEV may lead to morbidity and mortality in swans, geese, and wild waterfowl, and it is considered a serious threat to waterfowl species.^283^, ^284^, ^285^

Wu et al.^286^ examined the possible role of miR-30a-5p in DEV infection. They used qRT-PCR to measure Beclin-1 mRNA expression and levels of miRNAs (miR-30a-5p). Researchers used a dual-luciferase reporter assay (DLRA) to determine miR-30a-5p-Beclin-1 target interaction and western blotting to analyze Beclin-1 expression and autophagy in duck embryonic fibroblasts (DEFs). Moreover, the median tissue culture infective dose (TCID_50_) was calculated to estimate DEV titers. The results showed considerable downregulation of miR-30a-5p and remarkable upregulation of Beclin-1 mRNA in the DEV-infected DEFs. In addition, a DLRA verified the direct targeting of the 3′ UTR of the Beclin-1 gene by miR-30a-5p. Overexpression of miR-30a-5p decreased the level of Beclin-1 protein expression and Beclin-1-mediated autophagy, and it eventually repressed DEV replication. Alternatively, transfection of a miR-30a-5p inhibitor increased Beclin-1-mediated autophagy and stimulated DEV replication over the entire course of DEV infection. It was concluded that miR-30a-5p was capable of inhibiting DEV replication via reduction of autophagy by targeting Beclin-1. This approach could be a new anti-viral treatment strategy against DEV infection.^286^

### DENV and autophagy

Dengue is a common arthropod transmitted viral disease in tropical and subtropical areas of the world.^287^ In DENV infections, the patient can either suffer from dengue shock syndrome (DSS) and serious dengue hemorrhagic fever (DHF) or self-limiting dengue fever (DF).^287^ Researchers have considered cell autophagy to be necessary for efficient DENV replication.^116^^,^^288^, ^289^, ^290^ Autophagy is involved in DENV replication via alterations in cellular lipid metabolism that increase the generation of the ATP required for DENV replication.^291^ Most investigations have been carried out in liver cell lines, such as Huh7 and HepG2. However, other studies have given contradictory results, showing that the induction of autophagy decreased the DENV2 yield in the monocytic cell line U937.^292^ Consequently, the effects of autophagy on DENV replication appear to be mediated in a cell type-specific manner and require further work to understand the full implications.

miR-146a has already been shown to play a role in DENV replication inside monocytes via affecting the TRAF6 innate adaptor molecule.^293^ Pu et al.^294^ reported a new contribution of miRNA miR-146a to the negative regulation of cellular autophagy in DENV-infected A549 cells and THP-1 cells. Results showed that overexpression of miR-146a blocked DENV2-induced autophagy and, conversely, that locked nucleic acid (LNA)-mediated suppression of miR-146a abrogated these effects. In addition, overexpression of TRAF6, which is a target of miR-146a, reversed the suppressive effect of miR-146a on autophagy. Furthermore, treatment with recombinant IFN-β fully restored the autophagy activity in the TRAF6-silenced cells. In DENV2-infected A549 cells, autophagy enhanced the pro-inflammatory response with increased production of TNF-α and IL-6. It was concluded that miR-146a is a negative regulator of DENV-induced autophagy and that TRAF6 was a main target of this miRNA in the modulation of DENV autophagy.^294^

### Bovine viral diarrhea virus (BVDV) and autophagy

BVDV is an enveloped, positive sense ssRNA virus belonging to the genus *Pestivirus* in the Flaviviridae family.^295^ BVDV causes multiple diseases in cattle, such as hemorrhagic syndrome, diarrhea, respiratory disorders, reproductive disorders, mucosal disease, and persistent infections, which cause considerable economic losses to the cattle industry.^296^ Moreover, infection with the BVDV strain NADL considerably induced autophagy in Madin-Darby bovine kidney (MDBK) cells and enhanced the level of expression of two autophagy-associated genes, ATG14 and BECN1. In addition, the initial phases of autophagy were involved with BVDV NADL replication in MDBK cells.^297^

Fu et al. examined whether the *Bos taurus* (cattle) bta-miR-29b was upregulated in MDBK cells after infection with the BVDV strain NADL.^298^ Six hours after infection, the miR-29b level in infected cells was found to be 2.3-fold higher compared to normal cells. The autophagy process in BVDV NADL-infected cells was significantly inhibited by lentivirus-induced upregulation of miR-29b via direct targeting of ATG14 and ATG9A, two major autophagy-related proteins. The results were confirmed by overexpression of ATG14 and ATG9A leading to upregulation of autophagy and increased BVDV NADL replication. BVDV NADL replication was induced in MDBK cells during the early stages of autophagy, and it was inhibited after suppression of autophagy. It was concluded that there was a new connection between viral replication and miR-29b.^298^

## Indirect regulation of the anoikis pathway by cellular miRNAs after virus infection

As discussed above, once metastatic cancer cells are detached from the ECM, they become resistant to anoikis cell death, thereby penetrating into the lymph and blood circulation.^299^^,^^300^ Tropomyosin 1 (TPM1) has mostly been studied as a tumor suppressor gene, which inhibits the transformed phenotype and reduces anchorage-independent growth. In addition, TPM1 downregulation led to inhibition of anoikis in breast cancer cells.^301^, ^302^, ^303^ Interestingly, high molecular weight (HMW) TPM1 was downregulated by high levels of oncomir hsa-miR-2 in transformed cells.^304^^,^^305^ The HMW forms of TPM1 and TPM2 undergo translocation to the endothelial cell surface when activated by growth factors such as vascular endothelial cell growth factor (VEGF) or basic fibroblast growth factor (bFGF). A variety of ligands, including histidine-proline-rich glycoprotein (HPRG),^306^^,^^307^ cleaved HMW kininogen (HKa),^308^^,^^309^ and endostatin^310^ can then bind to TPMs on the cell surface, acting as their receptors. The anti-angiogenic effects of such binding can be abrogated by administration of engineered antibodies against the TPM1 and TPM2 cell surface receptors.^311^ Therefore, TPM1 can not only stabilize actin stress fibers in favor of anti-angiogenesis, but it can also promote anoikis^301^ and inhibit cancer development in primary breast tumor cells.^302^

### Kaposi’s sarcoma (KS)-associated herpesvirus (KSHV) and anoikis

KSHV causes the vascular hyperplasic disease KS by infecting endothelial cells.^52^^,^^312^^,^^313^ KSHV may also infect B lymphocytes and contribute to the multicentric lymphoproliferative condition called Castleman’s disease,^314^ or to primary effusion lymphoma.^315^ It was found that endothelial cells that have been infected by KSHV undergo malignant transformation with a higher angiogenic activity.^312^^,^^313^ KSHV has been shown to be in the latent phase in most KS cells, which only express a limited number of viral proteins, but there are 18 mature KSHV miRNAs derived from 12 precursor (pre-)miRNAs.^316^ Some targets of KSHV miRNAs (miR-Ks) have been identified.^317^, ^318^, ^319^ In the course of KS, a major rearrangement of the host cytoskeleton is observed,^320^ and two gene expression microarray assays were used to show that cytoskeleton TPM1 was downregulated in KSHV infection of telomerase-immortalized microvascular endothelial (TIME) cells or lymphatic endothelial cells (LECs).^321^^,^^322^ Because anoikis is inhibited by TPM1 downregulation in breast cancer cells,^301^ and also by KSHV-induced expression of vFLIP,^323^ it is proposed that KSHV-infected cells show partial resistance to anoikis cell death.

Kieffer-Kwon et al.^74^ examined whether the HMW TPM1 isoforms, which they showed to be downregulated by two KSHV miRNAs (miR-K2 and miR-K5), were able to suppress anoikis. They detected a functional binding site for miR-K5 in the 3′ UTR of one TPM1 isoform. Although KSHV-infected cells showed relatively higher levels of TPM1 protein compared to normal cells, these infected cells expressed even higher levels of TPM1, once miR-K2 or miR-K5 was downregulated. Mechanistically, miR-K2/miR-K5-induced downregulation of TPM1 isoforms increases VEGFA expression and tube formation from non-anchored human umbilical vein endothelial cells (HUVECs), and it increases the survival rate of HUVECs via suppressing anoikis. Overall, it was concluded that miR-K2 and miR-K5 were probably involved in the development of KSHV pathogenesis.^74^

### HBV and anoikis

HCC is a complex and heterogeneous disease caused by different risk factors, including viral infections. When HCC undergoes an invasive transformation, it leads to metastasis, post-surgical recurrence, and poor response rates to chemotherapy, with a corresponding poor prognosis and lower survival rate.^324^^,^^325^ HCC may be caused by HBV infection, because DNA repair, cell cycle progression, cellular adhesion, transcription modulation, signal transduction, and apoptotic pathways are all regulated by the small open reading frame of the HBV genome, HBV X protein (HBx).^326^, ^327^, ^328^, ^329^ HBV can activate cellular oncogenes, promote genome instability, regulate the host immune response, and facilitate other oncogenic processes, and therefore HBV is considered to be a major carcinogenic virus.^330^

A member of the serine protease inhibitor (serpin) superfamily, named mammary serine protease inhibitor (maspin) or serpin B5, is highly expressed in normal epithelial cells but is down-expressed in cancer cells such as HCC cells.^331^ The maspin level is negatively associated with angiogenesis, tumor growth, and tumor cell migration.^332^, ^333^, ^334^, ^335^ Maspin also upregulates integrin expression, thus promoting cell-fibronectin binding and reducing metastasis.^335^ Several studies have shown that maspin is downregulated in HCC cell lines;^336^, ^337^, ^338^, ^339^ however, the role of maspin in HCC development is not fully understood.

Chen et al.^75^ explored the pathway by which HBV-infected HCC cells could regulate maspin expression, as well as the secondary effects of this regulation. In accordance with previous studies, HCC cells exhibited a lower level of maspin expression. Mechanistically, miR-7, miR-21, and miR-107 were found to be upregulated by HBx, which, in turn, resulted in maspin downregulation. Moreover, analysis of patient samples showed a relationship between increased expression of miRNAs and maspin downregulation in HBV-infected HCC patients that was also linked to poor overall survival. The cancer cell resistance to anoikis, tumor metastasis, and chemoresistance were all enhanced by HBx-induced maspin downregulation. In addition, overexpression of maspin mediated by a lentivirus enhanced the sensitivity of the HBx-expressing HCC cells to anoikis, as well as increasing their viability. The data showed that downregulation of maspin via miRNAs is involved in the HBx-mediated malignancy and chemoresistance in HCC.^75^

## Conclusions

Viral pathogens are a major threat to public health, either by directly causing viral infections or being responsible for several cancer types. The underlying mechanism explaining how viruses affect different cellular functions is of great importance. One of the most critical cell functions affected by viruses is programmed cell death, which has repeatedly been shown to be mediated by several miRNAs. miRNA-dependent regulation of cell death pathways has proved to be a complex yet crucial issue, with more and more miRNAs having been found to be involved in both positive and negative regulation of these pathways. In this review, we summarized the available data on the virus-regulated miRNAs that modulate cell death pathways, and that can be used for further development of diagnostic and therapeutic techniques.

As shown in Table 1, several miRNAs, such as miR-155, miR-34a, miR-193b, and miR-93, have been reported to be regulated by various viruses. For instance, miR-21 was upregulated by several different viruses, such as HBV, EBV, and GaHV-2. Nevertheless, its target, the tumor suppressor PDCD4, was the same regardless of the virus type. However, it was reported that miR-21 regulated by the same HBV had different targets in each study (e.g., PDCD4, PTEN, IL-12, maspin, Fas-L). It can also be concluded that the same miRNAs often participate in the regulatory pathways that affect different types of cell death. For instance, the upregulation of miR-21 by HBV led to the inhibition of both apoptosis and anoikis. HBV led to the downregulation of miR-192-3p and the upregulation of the closely related miR-192-5p; the former enhanced autophagy while the latter inhibited apoptosis. This may be due to the presence of connections between different cell death pathways, which has already been shown. Most intriguingly, the regulation of cell death pathways by specific miRNAs was found to be different depending on the type of virus. For instance, miR-34a downregulation by HPV 16 and HPV 18 led to inhibition of apoptosis, whereas miR-34a downregulation by H1N1 produced the opposite result. In other words, apoptosis could result from both upregulation and downregulation of miR-34a, depending on the type of host cell and viral pathogen.

Overall, the miRNAs that are involved in the modulation of cell death pathways after virus infection show a degree of complexity and a variety of functions. Further studies are required to fully understand the complex pathways and functional targets before any therapeutic applications can be tested.

## Author contributions

H.M. was involved in the conception, design, statistical analysis, and drafting of the manuscript. N.R., L.S., S.A.A., M.M.-T., M.R.H., V.T., N.R., J.S.N., M.S.E., H.B.B., and H.R.M. contributed to data collection and manuscript drafting. M.R.H. critically edited the manuscript. All authors approved the final version of the manuscript for submission.

## Declaration of interests

The authors declare no competing interests.
